# Delayed Massive Hemothorax From Traumatic Inferior Pulmonary Ligament Tear: A Case Report

**DOI:** 10.1002/rcr2.70391

**Published:** 2025-10-28

**Authors:** Sachie Koike, Ryoichi Kondo, Kyoko Yamada

**Affiliations:** ^1^ Department of Thoracic Surgery NHO Matsumoto Medical Center Matsumoto Nagano Japan; ^2^ Division of General Thoracic Surgery, Department of Surgery Shinshu University School of Medicine Matsumoto Nagano Japan

**Keywords:** hemothorax, inferior pulmonary ligament, surgical treatment, traumatic, video‐assisted surgery

## Abstract

Hemothorax is often caused by blunt or penetrating trauma, a medical procedure, or direct tumoral erosion. Traumatic hemothorax is usually a consequence of intrathoracic organ damage, such as the chest wall or lung parenchyma. Herein we present a very rare case of traumatic hemothorax caused by inferior pulmonary ligament injury. The patient was a 64‐year‐old man, and he got injured due to loss of consciousness during bicycle riding. Since the patient was in a shock state and bleeding continued, video‐assisted thoracic surgery was performed. We found that a small laceration of the inferior pulmonary ligament was a cause of hemothorax and haemostasis with soft coagulation was performed. The inferior pulmonary ligament injury is an extremely rare condition as a cause of traumatic hemothorax, and only two cases including the present case have been reported. We should consider inferior pulmonary ligament injury as one of the causes of traumatic hemothorax.

## Graphical abstract

We present a very rare case of traumatic delayed hemothorax caused by inferior pulmonary ligament injury. The bleeding was from the small tear of the inferior pulmonary ligament.

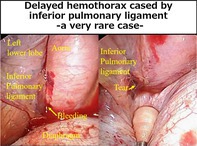

## 1 Introduction

Hemothorax is often caused by blunt or penetrating trauma, medical procedures or direct tumour erosion [1]. Traumatic hemothorax is usually a consequence of intrathoracic organ damage to the chest wall, lung parenchyma, tracheobronchial tree, oesophagus, diaphragm, heart or great vessels [2, 3]. Herein, we present a rare case of hemothorax due to inferior pulmonary ligament injury caused by blunt trauma.

## 2 Case Report

**FIGURE 1:**
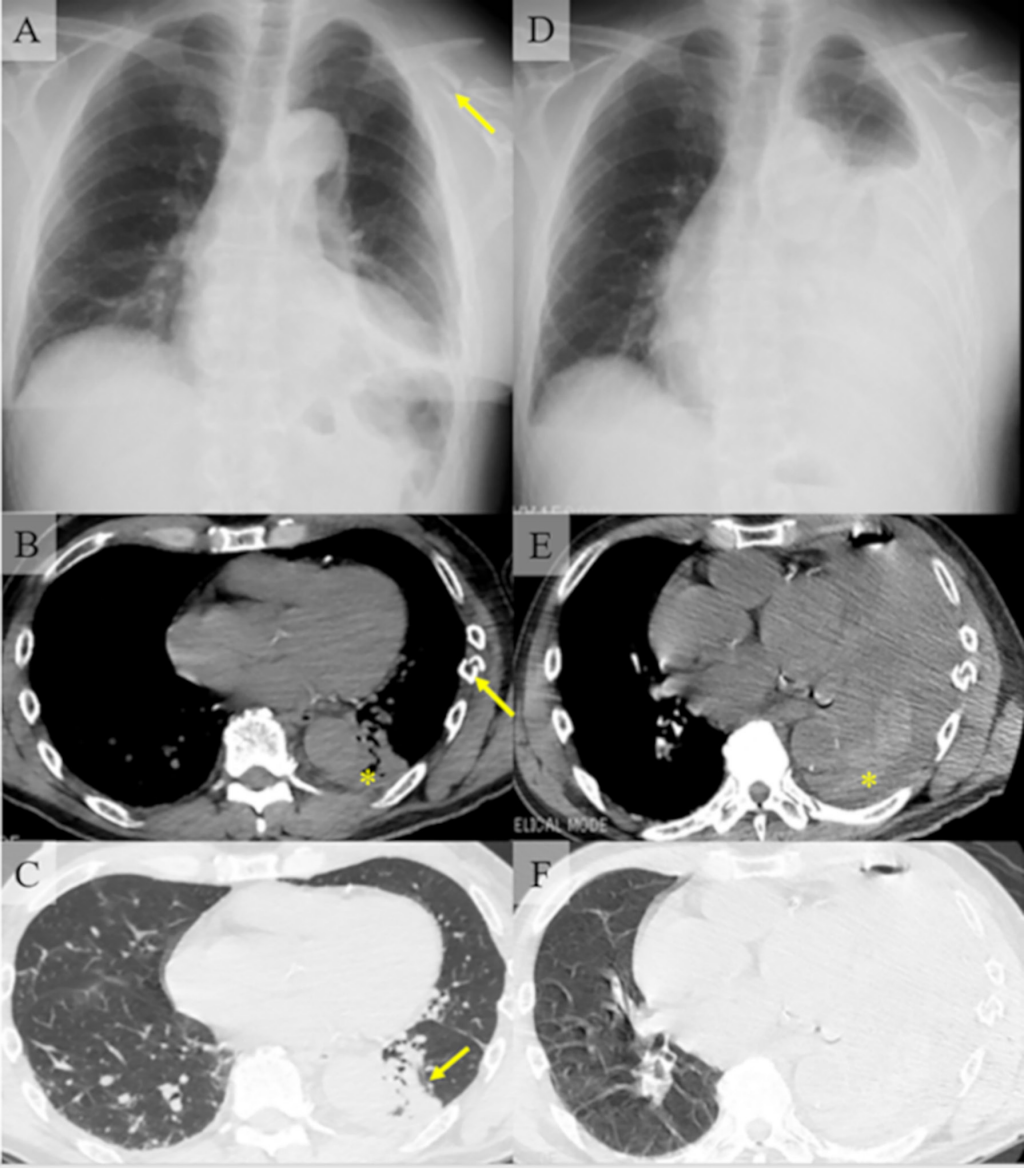
Radiological images of the patient at first visit (A–C). (A) Chest x‐ray revealed multiple rib fractures, a small amount of left pleural effusion, and a fracture of the left clavicle (arrow). (B) Chest computed tomography revealed fractures of the left 2nd to 8th ribs. This figure focused on the fracture of the 7th rib (arrow). A small amount of high‐density pleural effusion was also revealed (*). (C) A small contusion of the left lower lobe was revealed (arrow). Radiological images after vital signs changed (D–F). (D): A massive left pleural effusion was found. (E) A massive high‐density left pleural effusion was also observed (*). (F) Lung window setting of panel E.

A 64‐year‐old man with a history of paroxysmal atrial fibrillation (no anticoagulants were prescribed), chronic heart failure, and hyperlipidemia was referred to our hospital with multiple left rib and clavicle fractures. The patient had fallen off his bicycle due to a sudden loss of consciousness and was injured. The patient was hemodynamically stable. Chest x‐ray and computed tomography (CT) revealed fractures of the left 2nd to 8th ribs and clavicle, a small contusion of the left lower lobe, and a small amount of high‐density pleural effusion (Figure 1A–C). Multiple rib fractures, lung contusions, and hemothorax were planned for follow‐up at the outpatient clinic, and clavicle fractures were planned for surgical treatment by orthopaedic surgeons. On the day of the surgery, that is, 3 days after the injury, the patient had dyspnea and his oxygen saturation level was < 90%. His blood pressure was 88/65 mmHg, heart rate was 77 bpm, and respiratory rate was 24 breaths/min. The laboratory tests showed a decrease in haemoglobin levels (12.8–9.1 g/dL). Chest x‐ray and contrast‐enhanced CT revealed massive left pleural effusion. No obvious extravasation was observed (Figure 1D–F). Chest tube drainage was performed and 1900 mL of bloody pleural effusion was continuously drained from the chest tube. We diagnosed the patient with hypovolemic shock caused by massive hemothorax and decided to perform surgical treatment for haemostasis.

**FIGURE 2:**
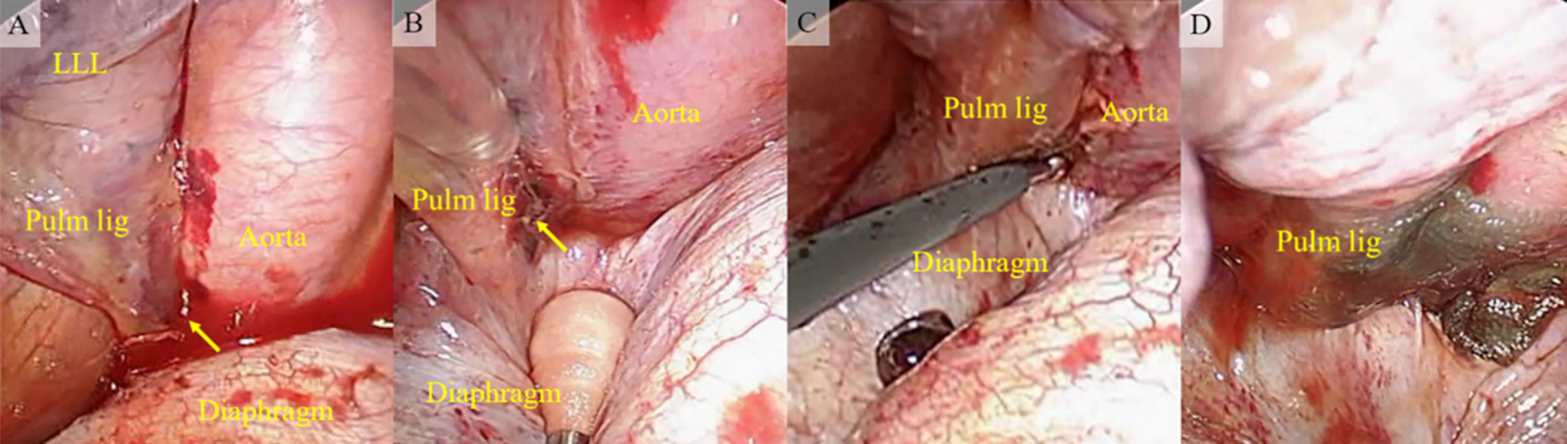
Intraoperative findings. (A) Bleeding from the inferior pulmonary ligament tear (arrow). (B) A small tear of the inferior pulmonary ligament (arrow) was found. (C) Haemostasis of the bleeding point was restored by soft‐coagulation electrocautery. D: Reinforcement was performed with polyglycolic acid sheets and fibrin glue. LLL = left lower lobe, Pulm lig = inferior pulmonary ligament.

Video‐assisted thoracic surgery (VATS) was performed with three skin incisions in the lateral decubitus position: a 3 cm camera port was placed on the VII intercostal space along the anterior axillary line, a 4 cm working port was placed on the VII intercostal space along the posterior axillary line, and the VIII intercostal space along the posterior axillary line. We found a 400 mL hemothorax with blood clots and removed it. We investigated the cause of the hemothorax and found exudative bleeding from a small tear in the inferior pulmonary ligament (Figure 2A,B). There were no signs of bleeding from the chest wall or lung parenchyma. Haemostasis was achieved using soft‐coagulation electrocautery (Figure 2C). Subsequently, the bleeding point was covered with polyglycolic acid (PGA) sheets and fibrin glue as reinforcement (Figure 2D). After the surgical treatment for haemostasis, surgery for the left clavicle fracture was performed by orthopaedic surgeons. Transfusion of two units of packed red cells was performed during the operation.

During the postoperative course, the patient became hemodynamically stable, and no signs of bleeding were detected. The laboratory tests showed an increase in haemoglobin levels (9.1–11.6 g/dL). The patient was discharged on postoperative day 9 without pulmonary complications.

## 3 Discussion

Traumatic hemothorax due to inferior pulmonary ligament injury is extremely rare, and only one case has been previously reported [3]. The inferior pulmonary ligament comprises a double membrane of pleura that drapes caudally from the lung root and loosely tethers the medial side of the lower lobe of the lung to the mediastinum. The space between the two membranes of the inferior pulmonary ligament contains small arterial branches from the bronchus and oesophagus, venules draining into the superior diaphragmatic veins, and lymphatic vessels emptying into the lower lobes of the lung and lower third of the thoracic oesophagus. These vessels become a source of bleeding when they are injured [3]. The inferior pulmonary ligament can be torn by a remarkable pressure gradient, such as traction or compression, because it anchors the lower lobe to the mediastinum and diaphragm [3].

In the present case, massive hemothorax with hypovolemic shock was observed 3 days after the injury. This suggests that bleeding from the small tear of the inferior pulmonary ligament continued for 3 days. We hypothesized that the main reason for this continuous bleeding was the mobility of the inferior pulmonary ligament. The inferior pulmonary ligament is a membranous structure that moves during breathing or body movements. The stabilisation of the bleeding point is an important factor in haemostasis. Surgical stabilisation of rib fractures has been reported to be an effective strategy for reducing the incidence of retained hemothorax [4]. The instability of the bleeding point in the inferior pulmonary ligament may prevent spontaneous haemostasis. The patient did not stay in bed and had a normal life at home after the injury, which may have exacerbated the condition.

VATS is the standard treatment for a retained hemothorax [2]. When VATS is performed early after injury, better outcomes have been reported regarding the length of hospital stay and morbidity compared with thoracotomy and conservative treatment [2]. In the present case, we performed VATS on the day of the hemothorax diagnosis, and the postoperative course was good. Because the inferior pulmonary ligament tear was small, we achieved less invasive haemostasis with soft‐coagulation electrocautery, PGA sheets and fibrin glue.

In conclusion, we report a rare type of delayed massive hemothorax caused by a traumatic inferior pulmonary ligament tear. An inferior pulmonary ligament injury should be considered as one of the causes of traumatic hemothorax, and its mobility may lead to continuous bleeding.

## Author Contributions

Conception and design: all authors. Collection and assembly of data: all authors. Manuscript writing: Sachie Koike and Ryoichi Kondo. Final approval of manuscript: all authors.

## Ethics Statement

This case report was approved by the Ethics Review Boards of NHO Matsumoto Medical Center, Matsumoto, Nagano, Japan (approval number:25‐09).

## Consent

The authors declare that written informed consent was obtained for the publication of this manuscript and accompanying images and attest that the form used to obtain consent from the patient complies with the Journal requirements as outlined in the author guidelines.

## Conflicts of Interest

The authors declare no conflicts of interest.

## Data Availability

The data that support the findings of this study are available on request from the corresponding author. The data are not publicly available due to privacy or ethical restrictions.

## References

[1] L. Huang , T. Pradhan , and O. Epelbaum , “Hemothorax Resulting From Hemorrhagic Degeneration of Giant Uterine Leiomyomata: A Rare Variant of Pseudo‐Meigs' Syndrome,” Respiratory Medicine Case Reports 55 (2025): 102207. 40452830 10.1016/j.rmcr.2025.102207 PMC12124651

[2] C. A. Beyer , A. C. Ruf , A. B. Alshawi , and J. W. Cannon , “Management of Traumatic Pneumothorax and Hemothorax,” Current Problems in Surgery 63 (2025): 101707. 39922629 10.1016/j.cpsurg.2024.101707

[3] J. J. Kim , Y. H. Kim , S. Y. Choi , S. C. Jeong , and S. W. Moon , “Life‐ Threatening Hemothorax due to the Inferior Pulmonary Ligament Injury Without Obvious Organ Injuries: a Case Report,” Journal of Cardiothoracic Surgery 10 (2015): 35. 25885049 10.1186/s13019-015-0243-8 PMC4369806

[4] S. Majercik , S. Vijayakumar , G. Olsen , et al., “Surgical Stabilization of Severe Rib Fractures Decreases Incidence of Retained Hemothorax and Empyema,” American Journal of Surgery 210 (2015): 1112–1116. 26454653 10.1016/j.amjsurg.2015.08.008

